# Effect of Gestational Weight Gain on Perinatal Outcomes in Low Risk Pregnancies with Normal Prepregnancy Body Mass Index

**DOI:** 10.1155/2019/3768601

**Published:** 2019-07-03

**Authors:** Mefkure Eraslan Sahin, Ilknur Col Madendag

**Affiliations:** Department of Obstetrics and Gynecology, Kayseri City Hospital, Kayseri, Turkey

## Abstract

**Objective:**

This study aimed to clarify the effect of gestational weight gain (GWG) on perinatal outcomes in low risk pregnancies with normal prepregnancy body mass index (BMI).

**Study Design:**

A total of 572 low-risk pregnant women with a normal prepregnancy BMI were included. GWG and inadequate or excessive weight gain were defined according to the United States Institute of Medicine updated guidelines. Adverse perinatal outcomes were compared among inadequate, normal, and excessive weight gain groups.

**Results:**

Of the 572 pregnant women enrolled, 62 belonged to inadequate GWG group, 80 to excessive GWG group, and 430 to normal GWG group. Maternal age, prepregnancy BMI, gravity, parity, and previous cesarean delivery rates were similar among groups. Adverse perinatal outcomes were not statistically significant among groups. Fetal weight was significantly lower in inadequate weight gain group compared to normal weight gain group (p<0.001) and fetal weight was significantly lower in normal weight gain group compared to excessive weight gain group (p<0.001). Additionally, low birth weight <2.5kgs, birth weight > 4.0kgs, and SGA and LGA rates were similar among groups (*P* = 0.765,* P* = 0. 711,* P* = 0. 702, and* P* = 0.414, respectively). Although gestational age at delivery was term in normal percentile it was significantly lower in the inadequate weight gain group compared to others (*P*=0.010).

**Conclusions:**

This study showed that an inadequate or excessive weight gain in low-risk pregnancies with a normal prepregnancy BMI did not increase the risk of adverse perinatal outcomes.

## 1. Introduction

In a routine obstetrics clinic, prenatal care providers are often asked the following question. How much weight should I gain during this pregnancy? In 2009, the United States Institute of Medicine (IOM) published updated guidelines to provide guidance on healthy gestational weight gain (GWG) expectancies for health care workers [1]. These guidelines are specific to the prepregnancy body mass index (BMI) and are intended to guide clinicians advising women on gestational weight gain.

In a literature it is well documented that prepregnancy obesity increases the risk of cesarean delivery, gestational diabetes, preeclampsia, abnormal fetal growth, and dystocia [2–4]. It is difficult to decide whether the prepregnancy BMI or excessive GWG is related with a major risk for adverse outcomes when excessive GWG occurs in women with obesity. It can be easily presumed that, independent of GWG, higher prepregnancy BMI is a major risk factor for both mother and fetus. Women with a prepregnancy normal BMI are more likely to deliver without adverse perinatal outcomes. Hence, this study aimed to determine the effect of GWG on perinatal outcomes in low risk pregnancies with normal prepregnancy BMI.

## 2. Materials and Methods

This retrospective cohort study was conducted at Sivas Sarkısla Government Hospital. The study was approved by the “Ethics Committee of Cumhuriyet University” (decision number: 2018-01/14) and performed according to the Declaration of Helsinki.

The study included pregnant women with a normal prepregnancy BMI (aged 18–35 years) who were followed up at the obstetric clinic between January 2015 and January 2018. The patients were divided into three groups according to GWG until delivery: (1) control, normal GWG; (2) inadequate GWG; and (3) excessive GWG. Adverse perinatal outcomes were compared among women with inadequate weight gain (*n*=62), excessive weight gain (*n*=80), and normal weight gain (*n*=430).

Patients with multiple pregnancy, type 1 or 2 diabetes, personal gestational diabetes history, chronic hypertension, personal gestational hypertension or preeclampsia history, intrauterin growth restriction(IUGR), systemic chronic disease, congenital or chromosomal abnormalities, and smoking, alcohol, or drug use were excluded from the study. Additionally, underweight, overweight, and obese patients were excluded according to the prepregnancy BMI.

While the BMI was calculated as prepregnancy weight in kilograms by area in meters, GWG was defined as the difference between the actual weight at birth and the initial weight before conception. The BMI was classified according to the values determined by the World Health Organization (underweight,<18.5 kg/m^2^; normal weight, 18.5–24.9 kg/m^2^; overweight, 25–29.9 kg/m^2^; and obese >30 kg/m^2^) [5].

Values for the GWG under the recommended range, within this range, and over this range were compared using the 2009 IOM guidelines for each pre-pregnancy BMI category [1]. Women with a GWG in the range recommended by the IOM were classified as having a normal GWG. In this guidline, recommended total weight gain during pregnancy was classified for patients' prepregnancy BMI levels. Accordingly, weight gain of 11.5-16.0kg (20.0-350 lbs) during pregnancy is considered normal for pregnant women with normal BMI before pregnancy. In the present study, weight gain of less than 11.5 kg was defined as inadequate and weight gain of more than 16.0 kg was defined as excessive weight gain. According to IOM recommendations, those with inadequate or excessive GWG were classified as those with less or excessive GWG, respectively. In routine clinical practice we prefered delivery induction as the presence of any of the following: oligohydramnios after 39 weeks of gestation, anhydramnios after 34 weeks of gestation, and premature rupture of membranes after 34 weeks of gestation.

Adverse perinatal outcomes were defined as the presence of any of the following: prematurity (delivery before 37 weeks of gestation), Apgar 5 min <7, cesarean delivery for nonreassuring fetal heart-rate testing, transient tachypnea of the newborn (TTN), respiratory distress syndrome (RDS), meconium-stained amniotic fluid, and hyperbilirubinemia. Additionaly low birth weight <2.5kgs, birth weight > 4.0kgs, small for gestational age (SGA), and large for gestational age (LGA) rates were compared among groups.

The comparison of more than two groups was investigated using ANOVA followed by Tukey's post hoc test with Minitab 16 (Minitab Inc.; State College, PA, USA). The difference between groups was considered statistically significant when p value was <0.05

## 3. Results

Of the 572 pregnant women enrolled in the study, 62 belonged to the inadequate GWG group, 80 to the excessive GWG group, and 430 to the normal GWG group. Their demographic and obstetric characteristics were compared and are shown in Table 1. Maternal age, prepregnancy BMI, gravity, parity, and previous cesarean delivery rates were similar in both groups.

Table 2 shows the adverse perinatal outcomes. Male sex, induction of labor, prematurity, cesarean delivery for nonreassuring fetal heart-rate testing, Apgar 5 min <7, TTN, RDS, meconium-stained amniotic fluid, and hyperbilirubinemia rates were not statistically different among groups (*P* = 0.887,* P* = 0.571,* P* = 0.868,* P* = 0.881,* P* = 0.870,* P* = 0.988,* P* = 0.864,* P* = 0.841,* P* = 0.386, respectively). Fetal weight was significantly lower in inadequate weight gain group compared to normal weight gain group (p<0.001) and fetal weight was significantly lower in normal weight gain group compared to excessive weight gain group (p<0.001). Additionaly, low birth weight <2.5kgs, birth weight > 4.0kgs, and SGA and LGA rates were similar among groups (*P* = 0.765,* P* = 0. 711,* P* = 0. 702, and* P* = 0.414, respectively). Although gestational age at delivery was term in normal percentile it was significantly lower in the inadequate weight gain group compared to others (*P*=0.010).

## 4. Discussion

The present study showed that an inadequate or excessive weight gain in low risk pregnancies with a normal prepregnancy BMI was not found to increase the risk of adverse perinatal outcomes.

Various studies in the literature evaluated GWG and adverse perinatal outcomes. Karen E. Hannaford et al. reported that an inadequate weight gain led to 2.5 times more risk for SGA and 2 times more risk for preterm delivery [6]. Chunming Li et al. confirmed that individuals who weighed less than that recommended by the IOM were twice as likely to have an SGA-born baby as the normal-weight group [7]. These results were consistent with the findings of Ricci E. et al. [8]. Oken E. et al. stated that the weight gain higher than that recommended by the IOM was associated with macrosomia, high birth weight, and LGA babies [9]. Another study showed that weight gain higher than that recommended by the guidelines was associated with the birth of LGA newborns [10, 11]. These studies had different inclusion criteria, especially underweight, overweight, and obese prepregnancy BMI, advanced age (≥35 years), smoking, and personal history of gestational diabetes or gestational hypertension. Therefore, potential risks associated with an inadequate or excessive weight gain in low-risk pregnancies with a normal prepregnancy BMI are not clear.

The results of the present study indicated that an inadequate or excessive weight gain in low-risk pregnancies with a normal prepregnancy BMI was not a risk factor for adverse perinatal outcomes. The positive results could be explained by the absence of risk factors. Shin, Dayeon, et al. reported that the prepregnancy BMI was an independent risk factor for gestational diabetes, gestational hypertension, preterm delivery, and delivery of SGA or LGA babies [12]. Mei-Dan E. et al. demonstrated that preterm delivery, lower birth weight, RDS, and neonatal mortality rates were significantly higher in the active smoking group than control group [13]. In addition, Lıou, Jui-Der, et al. reported that advancing age was associated with operative vaginal birth, cesarean delivery, premature delivery, early preterm birth (before the 34th gestational week), fetal death, low Apgar scores, birth weight <1500 g, and neonatal death [14]. Similarly, Marozio, Luca, et al. showed that a maternal age more than 40 years was an independent risk factor for adverse maternal outcomes [15]. Isabel Fridmann et al. suggested that any weight change during a given gestation period, apart from the generally recommended weight, did not increase infant mortality [16]. Similarly, the preconception normal BMI category generally had the lowest infant mortality risk compared with women of all other BMI classes [17].

The study has some limitations. The retrospective design of the study, the low number of patients, and the lack of data on gastrointestinal problems, especially in the inadequate weight gain group, are important limitations.

An important finding of the present study was that the prematernal BMI was an important risk factor for neonatal outcomes. Hence, the reason for reducing the weight to an acceptable weight of gestation was to avoid all metabolic and birth complications that could affect the mother and the newborn. However, nearly half of pregnancies are unplanned, and overweight and obese women may not have the ability to lose weight before conception. Therefore, these women should be monitored more closely by obstetricians to minimize the negative consequences of overweight during pregnancy.

## 5. Conclusion

In conclusion, the present study showed that an inadequate or excessive weight gain in low-risk pregnancies with a normal prepregnancy BMI did not increase the risk of adverse perinatal outcomes. Further prospective studies with a larger number of patients are required in this regard.

## Figures and Tables

**Table 1:** Comparison of demographic and obstetric characteristics between groups.

	Inadequate weight gain (n:62)	Normal weight gain (n:430)	Excessive weight gain (n:80)	p-value
Maternal age (years)	29.1±3.1	28.6±3.2	28.7±3.2	0.186
Pregestational BMI (kg/m^2^)	23.5±1.4	23.6±1.3	23.6±1.3	0.791
Gravity	2.5±0.8	2.4±0.7	2.5±0.7	0.548
Parity	2.0±0.7	2.1±0.7	2.1±0.7	0.326
Previous cesarean history (n%)	15 (24,1%)	99(23,2%)	19(23,7%)	0.971

The comparison of more than two groups was investigated using ANOVA followed by Tukey's post hoc test with Minitab 16 (MinitabInc.; StateCollege, PA, USA). The difference between groups was considered statistically significant when p value was <0.05.

**Table 2:** Comparison of perinatal outcomes among groups.

	Inadequate weight gain (n:62)	Normal weight gain (n:430)	Excessive weight gain (n:80)	p-value
Gestational age at delivery (week)	37,95±1,33^a^	38,50±1,33^b^	38,47±1,53^b^	*0.010*
Fetal weight (g)	3063±279^a^	3395±321^b^	3652±324^c^	*<0.001*
Low birth weight <2.5kgs (n%)	4 (6,4%)	22 (5,1%)	3 (3,7%)	0.765
Birth weight > 4.0kgs (n%)	2 (3,2%)	21 (4,8%)	5 (6,2%)	0.711
SGA (n%)	6 (9,6%)	30 (6,9%)	5 (6,2%)	0.702
LGA (n%)	2 (3,2%)	28 (6,5%)	7 (8,7%)	0.414
Male sex (n%)	34 (54,8%)	223(51,8%)	41(51,2%)	0.887
Induction of labor (n%)	13 (20,9%)	77 (19,0%)	19(23,7%)	0.571
Delivery<37 weeks (n%)	5 (8,0%)	35 (8,1%)	5 (6,2%)	0.868
Cesarean delivery for nonreassuring fetal heart rate testing (n%)	3 (4,8%)	17 (3,9%)	4 (5%)	0.881
Meconium-stained amniotic fluid (n%)	4 (6,4%)	25(5,8%)	7 (8,7%)	0.841
TTN (n%)	3 (4,8%)	20 (4,6%)	4 (5%)	0.988
RDS (n%)	1 (1,6%)	4 (0,9%)	1 (1,25%)	0.864
Hyperbilirubinemia(n%)	2 (3,2%)	7 (1,6%)	3 (3,6%)	0.386

*SGA: small for gestational age*, *LGA: large for gestational age*, *TTN*: transient tachypnea of the newborn, and *RDS*: respiratory distress syndrome.

*Note.* Different superscripts indicate statistically significant differences. Fetal weight was significantly lower in inadequate weight gain group compared to normal weight gain group (p<0.001). Fetal weight was significantly lower in normal weight gain group compared to excessive weight gain group (p<0.001).

The comparison of more than two groups was investigated using ANOVA followed by Tukey's post hoc test with Minitab 16 (MinitabInc.; StateCollege, PA, USA). The difference between groups was considered statistically significant when p value was<0.05.

## Data Availability

The data used to support the findings of this study are available from the corresponding author upon request.

## References

[1] Rasmussen K. M., Yaktine A. L., Institute of M (2009). National research council committee to reexamine IOMPWG. the national academies collection: reports funded by national institutes of health. *Weight Gain During Pregnancy: Reexamining the Guidelines. Washington (DC): National Academies Press (US)National Academy of Sciences*.

[2] Gunatilake R. P., Perlow J. H. (2011). Obesity and pregnancy: clinical management of the obese gravida. *American Journal of Obstetrics and Gynecology*.

[3] Siega-Riz A. M., Viswanathan M., Moos M. (2009). A systematic review of outcomes of maternal weight gain according to the institute of medicine recommendations: birthweight, fetal growth, and postpartum weight retention. *American Journal of Obstetrics & Gynecology*.

[4] Cosson E., Cussac-Pillegand C., Benbara A. (2016). Pregnancy adverse outcomes related to pregravid body mass index and gestational weight gain, according to the presence or not of gestational diabetes mellitus: a retrospective observational study. *Diabetes & Metabolism*.

[5] World Health Organization technical report series (1995). Physical status: the use and interpretation of anthropometry. *Report of a WHO Expert Committee*.

[6] Hannaford K. E., Tuuli M. G., Odibo L. (2017). Gestational weight gain: association with adverse pregnancy outcomes. *American Journal of Perinatology*.

[7] Li C., Liu Y., Zhang W. (2015). Joint and independent associations of gestational weight gain and pre-pregnancy body mass Index with outcomes of pregnancy in chinese women: a retrospective cohort study. *PLoS ONE*.

[8] Ricci E., Parazzini F., Chiaffarino F., Cipriani S., Polverino G. (2010). Pre-pregnancy body mass index, maternal weight gain during pregnancy and risk of small-for-gestational age birth: results from a casecontrol study in Italy. *The Journal of Maternal-Fetal and Neonatal Medicine*.

[9] Oken E., Kleinman K. P., Belfort M. B., Hammitt J. K., Gillman M. W. (2009). Associations of gestational weight gain with short- and longer-term maternal and child health outcomes. *American Journal of Epidemiology*.

[10] Li N., Liu E., Guo J. (2013). Maternal prepregnancy body mass index and gestational weight gain on offspring overweight in early infancy. *PLoS ONE*.

[11] Vesco K. K., Sharma A. J., Dietz P. M. (2011). Newborn Size among obese women with weight gain outside the 2009 institute of medicine recommendation. *Obstetrics & Gynecology*.

[12] Shin D., Song W. O. (2014). Prepregnancy body mass index is an independent risk factor for gestational hypertension, gestational diabetes, preterm labor, and small- and large-for-gestational-age infants. *The Journal of Maternal-Fetal & Neonatal Medicine*.

[13] Mei-Dan E., Walfisch A., Weisz B. (2015). The unborn smoker: association between smoking during pregnancy and adverse perinatal outcomes. *Journal of Perinatal Medicine*.

[14] Hsieh T. T., Liou J. D., Hsu J. J. (2010). Advanced maternal age and adverse perinatal outcomes in an Asian population. *European Journal of Obstetrics & Gynecology and Reproductive Biology*.

[15] Marozio L., Picardo E., Filippini C. (2017). Maternal age over 40 years and pregnancy outcome: a hospital-based survey. *The Journal of Maternal-Fetal & Neonatal Medicine*.

[16] Friedmann I., Balayla J. (2017). Gestational weight gain and the risk of infant mortality amongst women with normal prepregnancy BMI: the Friedmann-balayla model. *The Journal of Maternal-Fetal & Neonatal Medicine*.

[17] Houde M., Dahdouh E. M., Mongrain V., Dubuc E., Francoeur D., Balayla J. (2015). The effect of adequate gestational weight gain among adolescents relative to adults of equivalent body mass index and the risk of preterm birth, cesarean delivery, and low birth weight. *Journal of Pediatric & Adolescent Gynecology*.

